# Evaluation of spatial analysis application for urban emergency management

**DOI:** 10.1186/s40064-016-3723-y

**Published:** 2016-12-07

**Authors:** Rifaat Abdalla

**Affiliations:** Department of Hydrographic Surveying, Faculty of Maritime Studies, King Abdulaziz University, P. O. Box 80401, Jeddah, 21589 Kingdom of Saudi Arabia

**Keywords:** GIS, Emergency management, Urban centers, Spatial analysis, Environmental modeling

## Abstract

**Background:**

This paper provides multidisciplinary scope to the utilization of geospatial data frameworks for urban disaster management with accentuation on particular events. The emergency management events presented in this review are universally known and represent high risk for different parts of the world.

**Results:**

The discussion starts with addressing the application issues related to how spatial analysis can be used intending to disaster management operations by characterizing its ease of use and impediments in managing the inquiries of vulnerability and hazard assessment. It also highlights best practices for the approaches to integrating spatial data for hazard mapping and risk perception.

**Conclusions:**

The goal of this study is to give conceptual coverage to appropriate solutions for emergency preparedness and response, using spatial analysis and GIS. The paper emphasized that among different issues that may confront the use of spatial analysis, is the accuracy of data and time of processing, in addition to collective coordination of stakeholders working in the field. The findings of this research conclude that a challenge to possible risk reduction is furnishing disaster managers with access to information and methodologies that may help them in analyzing, evaluating and mapping hazard models.

## Background

GIS applications in Disaster Management are progressively turning into a necessary component of disaster and emergency management activities in many parts of the world. The time considerations are extremely critical in emergency management operations. Emergency Managers are required to take significant decisions, promptly to provide fast response to extreme situations. The spatial dimension of geospatial data makes it exceptionally critical for decision-makers in the different phases of emergency management operations. It is important for policy makers to have the right information at the ideal time exhibited off base models to permit them to react, arrange or moderate catastrophes. The temporal nature of disasters does not allow emergency managers to gather the critical data, in a timely, in many situations. As such, more often, pre-arranged disaster management scenarios are utilized Becerra-Fernandez et al. (2008). GIS Technology is capable of filling up the gap of perception and investigation of simulating emergency scenarios showing various situations and their temporal attributes. This permits disaster managers to have access to sufficient data stored in spatial databases and exhibited in a PC created maps or intuitive models Miura et al. (2007). GIS can be exceptionally useful to make well-thought counter disaster response patterns, which can address the overall population. It is a helpful tool in disaster management planning, tabletop activities, and a fundamental element of Emergency Operations Centers (EOC) (ESRI 1999). GIS gives a component to perception and demonstrating of primary data different levels of details and for various regions after a disaster strikes (ESRI 1999). This provides a user-driven approach, which envelops the phases of disaster management, to bolster the procedure of improved primary leadership and builds the level of inclusion of every group of workforce related exercises and systematic methods (Smirnov et al. 2006).

Discussion about disaster management is tending to the issues of preparedness. This is a crucial part of disaster and emergency management and can assume an indispensable part if contingency activities, which gets to be vital. The convenience of GIS as a decision support system is in helping disaster managers and emergency first responders to falls in the following:

1. Risk and Threads Assessment.
2. What-if scenario modeling.
3. Maintaining situational awareness.
4. Allocation of Resources and documenting disruptions.
5. Alerting and notification of communities.
6. Minimizing vital service disruptions during the response stage.

Saadatseresht et al. (2009) have presented the factors above are especially of significance from spatial analysis point of view In an emergency management situation. It showed that spatial analysis can be performed for emergency management evacuation operations, in order to relocated population at risk for a safer location, this is usually a complicated process, dense population. Anjum et al. (2011) indicated that it is important to use utilize the state-of-the-art of spatial analysis tools for emergency planning operations, related to evacuation of masses, during extreme events. A major challenge for using spatial analysis as a part of search and rescue plans is in deciding the evacuation process to relocate the evacuees to a safer place. This indicates that supporting the choice of where and from which street every evacuee ought to go is an essential factor in the best utilization of spatial analysis capabilities (Cova and Church 1997). Several factors are involved in determining the efficiency of utilizing the process of spatial analysis for disaster management. To adequately accomplish the point of spatial investigation in crisis administration operations, a few goals are brought into thought and fulfilled at the same time through this paper. These objectives are (a) how a decision-maker can utilize the effectiveness of spatial analysis for prioritizing important decisions, during an emergency. (b) What are essential capacities that spatial analysis can help with amid disaster management cycle in the ten chosen disaster themes focused in this paper? (c) How decision-makers could better actualize spatial analysis process as a significant aspect of their everyday operations.

Successful disaster management calls for including multi-modal decision-making competencies; that includes aggregates at all levels of relief and response, notwithstanding total relief endeavors that address the origin of vulnerability. Morrow (1999) the vulnerability of group is correctly credited to the socioeconomic variables that influence the group, i.e., the directly affected, whether expanded or diminished as a consequence of the socio-economic well-being of a community, as it identifies with their everyday practice (ESRI 1999). Emergency Management planners, policy makers, risk analysts and first responders usually attempt to characterize and find high-risk factors utilizing Community Vulnerability Maps, consolidating this information into GIS frameworks, and for this, spatial analysis is essential (Kumar 2013).

## Emergency management operations

Comprehensive Emergency Management (CEM) is a concept that ensures the effectiveness of all aspects of emergency management by anticipating, minimizing the risks introduced by various emergencies, by preparing for emergency situations, and by helping in recovering from an emergencies. This approach is systematically addressed by Gordon (2002), where he provided a framework for comprehensive approach for dealing with risks.

According to Bullock et al. (2006), the impact of disaster can impact every community, state or authorities within the proximity of the event. As such, there is no person immune to the impact of disasters. According to Mileti (1999) many disaster losses are to some degree predictable, which make them manageable to a certain extent. Effective processes in disaster management help to reduce devastation and high costs at both the local and regional levels. A comprehensive emergency management system is composed of the interaction of policies and procedures, as well as the institutional and financial mechanisms, to constitute community-based approach to disaster risk management (Carter 1991).

## Urban emergency management

This part covers a critical review on the use of spatial analysis in some urban emergency management situations. It provides an inside and out scope of the work cited in this regard to in such manner that it highlights the process of enhanced decision-making process. The extent of the scope will concentrate on the most important progressions in the utilization of spatial analysis methods for emergency management in urban situations.

### Spatial analysis applications in natural hazards

#### Earthquakes and humanitarian coordination

The literature on GIS and humanitarian coordination has started by first looking at the different approaches in which GIS can be utilized for effective coordination. Regardless of the way that GIS has been predominately seen, within the disaster management community, as a cartographic tool, an approach to managing initial analysis and visualization, or an electronic navigational system, this does not attractively depict the best way of GIS utilization in humanitarian assistance (Currion 2006). There are numerous potential utilizations of GIS for humanitarian aid. For instance, the usage of enhancement, which is the use of cutting edge GIS calculations to take care of an outline issue, can be utilized to discover reasonable areas for clearing. For example, a support investigation, for analysis of spatial relationships using GIS as a tool, can be employed to gauge vulnerability to various hazards based on proximity.

In 2005, a Complex Humanitarian Emergencies Study by Verjee (2005) drew from contextual analyses and examples in innovative progression to format the potential GIS applications for humanitarian emergencies, which were:

1. Mapping and Cartography (Land use Mapping, Infrastructure Mapping, Demographic Mapping, Logistics, and Sustainability).
2. Outreach, Media and Communications (Public Access to Information, Reporting, Program Assessment, News Coverage).
3. Modeling and Simulation for Disaster Scenarios (Practice, drills, and exercises, Data information flow, planning for contingencies).
4. Environmental Management and Planning (Planning, Yield Cultivation, resources assessment).
5. Risk and Hazard Management (seismic analysis, site selection and planning, and water level estimation and mitigation).
6. Vulnerability Analysis and Assessment (Early Warning frameworks for the dry season, desertification and starvation, Epidemics modeling and Tsunami Planning).
7. Risk Reduction (‘problem areas’ distinguishing proof and relief programming).
8. Response Policies and Organizational Management (administration, planning, and training).

Table 1 is demonstrating the capabilities of GIS in this situation. In spite of the fact that there are various applications of spatial analysis as a GIS technique, they all share an ultimate target, which is to which is to exploit the situational the situational awareness to all areas taking an interest so fundamental concerns can be perceived and after that together achieved.

**Table 1:** Applications of spatial analysis in humanitarian coordination

GIS application	Description of application	Tools	Application examples
*Queries and measurements*			
Queries	Does not change the database or produce new data but reveals information	Scatter plots, residuals, and structured/standard query language	To determine water sources near potential IDP settlements
Measurements	Numerical interrogations of GIS, which make an analysis of spatial data	Makeup distance, area, length, shape, aspect of spatial data	To estimate the total area needed to set up IDP settlements To determine the length between food distribution points
*Transformations*			
Transformations	Creates new data from existing data	Buffer analysis	Used to comply with Sphere humanitarian standards. For example, can be used to ensure that toilets are within 50 meters of shelters
Spatial interpolation	A transformation analysis method used for intelligently guessing the value of the discreet object	Thiessen polygons, inverse-distance weighting, and Kriging	To predict infection rates in IDP settlements To estimate flow rates of well to large aquifer
*Optimization*			
Point optimization	Determines optimal locations amongst nodes of a network	Network analysis	To select location for critical infrastructure such as food storage areas
Route optimization	Determines optimal routes amongst nodes of a network	Network analysis	To access accessibility of road networks to potential IDP settlements
Path optimization	Solves are routing problems by minimizing friction value and does not require a network	Network analyst	To determine paths for utility infrastructure for IDP settlements
*Analysis*			
Geostatistical analysis	Used to measure geographic distributions, identify pattern and clusters, and analyze geographic relationships	Geostatistical analysis	To determine historical rainfall averages over a particular area, which could be applied to potential IDP locations
Centroid analysis	Used to identify trends over various phenomena in a set period	Spatial analyst	To determine trends in infectious diseases and household income levels within IDP settlements
Pattern analysis	Used to identify distribution points. Can determine whether the point is random, clustered or dispersed	Spatial analyst	To identify links between water wells and infectious diseases
Relationship analysis	Used to determine the relationship between various geographic phenomena	ArcGIS 3D analyst, ArcGIS spatial analyst and ArcGIS network analyst	To assist in increasing the efficiency of daily operation of IDP settlements
Geovisualization	Simple three dimensions (3D) and high-resolution mapping are used globally to access a variety of information	Google earth, ESRI’s arcexplorer, and microsoft virtual earth	Allows a layperson to understand the unfolding of humanitarian emergency

A late analysis by Eveleigh et al. (2007) and Al-Ahmadi et al. (2014) has utilized spatial analysis for earthquake disaster studies. The adopted approach recognizes that within the scope of humanitarian assistance “GIS innovation is battling with how to address complex issues that require the modeling of rapidly changing dynamic phenomena, feature, behavior, data and. They concluded that there is a high potential for GIS-based assessment models to give the leap forward expected to address the random way of humanitarian emergencies.

Bally et al. (2005) presented the use of remote sensing for Humanitarian Aid, showing that the utilization of remote detecting and GIS permitted 200,000 IDPS to be migrated to longer-term settlements that had a renewable water source and with improvement potential in regards to sanitation, farming, and even hydropower. Another powerful GIS application used to support humanitarian emergencies was The Global Connection Project, which included Carnegie Mellon University, NASA, Google and National Geographic, contributing to the relief planning for October 8, 2005, South Asian earthquake and tsunami. In this project, GIS was utilized to gain and convey high-resolution imagery from Digital Globe’s Quickbird.

#### Wild fire

ESRI (1999) has shown an approach to depicting a rapidly spreading fire event precisely; spatial analysis can be utilized to recognize high-risk fire zones and set up buffer zones for evacuation. Notwithstanding the determination of high-risk regions, spatial analysis can be combined with statistical analysis as a verification method for the specifying areas of final damage assessment, in addition to deciding to provide visual models for highly impacted areas, according to Goodchild (2006). Lentile et al. (2006) gave direction by distinguishing potential layers that can be utilized for urban fire identification. The initial step was to employ scope and longitude directions to plot the different flames (based upon a decision of lightning or human-ignited fire) during a particular period. Fire information may seem, by all accounts, to be situated inside waterways. However, this is mainly a reason for adjusting buffer zones to give some slack to such errors. The process of connecting attributes information and present four analytical techniques for simulation and visualization out of control fire. In spite of the fact that their emphasis particularly on human-brought ablaze catastrophes, in proposing the four prescribed alternatives for finish urban fire examination:

- the territory influenced
- temporal expand
- spatial extend
- probability

The urban fire hazard is hard to avert. Notwithstanding, through the recognizable specification of the high-risk zones, the recurrence of flame can be minimized. Jaiswal et al. (2002) have demonstrated that GIS when joined with satellite imagery, can be useful in identifying high-hazard regions within given vicinity and restrict the fire spread and thus minimize the impact. Jaiswal et al. (2002) have also examined the utilization of ArcGIS for this idea, declaring that the mix of topographic foundation data and remote sensing for vegetation mapping can make a precise estimation of high-risk fire territories utilized for moderation and reaction purposes. In Jaiswal et al. (2002) different layers of vegetation, slope, proximity to settlements, and distance from roads were made to provide an indication about high-risk fire regions. After this data was plotted, buffer zones of 1000, 2000, 3000, and 4000 m surrounding the high-risk zones were plotted to extend the distinctive levels of danger. Although they have investigated a particular instance of India, the concept of using GIS spatial analysis consolidated with satellite imagery for distinguishing areas prone to high-risk of fire hazard has demonstrated the adequacy of GIS as a tool for urban disaster management. If GIS can be utilized to model and simulate high-risk fire zones with buffers, which gives benchmark understanding that GIS could likewise be used to show damage assessment models using different software and different data layers, regardless of the geographic location.

Pradhan et al. (2007) utilized GIS examination to decide fire susceptibility, using a “vector spatial database” with GIS and consolidated with topographic information, fuel information, base overview focuses, and maps. This took into account figuring variables, which were then changed over to a raster grid, recognizing 112 cells inside the fire events. A frequency-based proportion approach was used to characterize the “connections between hotspot areas and the components in the study area”. The challenges, notwithstanding, were in processing “a significant amount of data”. The conclusion is drawn from Pradhan et al. (2007) on the utilization of such projections for fire risk mapping and mitigation was quite compelling. In foreseeing fire susceptibility when utilizing frequency analysis, the prescribed results were recommended to be used with alert, according to Pradhan et al. (2007). It was suggested that the analysis approaches their examination is used fundamentally amid fire event, which proposes mapping fire-influenced zones instead of driving toward the relief bit of fire disaster management process.

#### Floods

Correia et al. (1998) demonstrated that GIS had been seen as a successful tool to organize and visualize data from different sources on far-reaching floodplain administration. As a part of this overall approach to manage floodplain management, it is crucial to have the ability to predict the aftereffects of different situations as to flooded regions and related regions at risk. Morrow (1999) discussed the hydrologic and water controlled zones accept a crucial part, and there is much to get in uniting these exhibiting capacities in a GIS system. The perspective of the using Intergraph GIS with IDRISI GIS provided an effective way in dealing with flood emergencies in both 2D and 3D. Using multidimensional modeling usually extended the flexibility of using GIS as an instrument for flood modeling. Gogoaşe Nistoran et al. (2016) have shown the effectiveness of spatial analysis using GIS for modeling flood inundation as a result of dam-break.

The role of GIS in Flood Disaster Management was analyzed by Cova (1999), through the perspective of Comprehensive Emergency Management (CEM) and its four phases: mitigation, preparedness, response and recovery. In the wake of a disaster, GIS is getting the chance to be vital in supporting damage assessment, evaluation, and cost estimation for development. In the aftermath of a catastrophe, GIS is a valuable tool in supporting cost evaluation and rebuilding. Abbas et al. (2009) proposed a GIS-based contemplate regarding the change of surge showing and representation for Allahabad Sadar Sub-District (India). This joins the framework, the methodology/approach that planned to research the degree for spatial analysis application for a rapid response. The flood affected zones have been recognized, and their positions are checked, where the GIS handiness has been manhandled to get the spatial information for the fruitful calamity organization for surge affected reaches. The adopted approach has helped in recognizing issues that may upgrade the present practices of emergency management organizations. The approach gives a suitable and quick fundamental authority instrument for snappy response to emergencies if used appropriately, which along these lines would help in minimizing loss of life and property. Al-Sabhan et al. (2003) proposed a GIS-construct study, in light of the change of flood levels and representation. This consolidates the arrangement, the investigated the present status of progressing hydrological models used for flood modeling and risk mitigation. It indicated how electronic systems could overcome a bit of the obstruction of existing structures. While hydrological GIS-based models are open, they are ineffectually suited to the consistent application and are frequently not primarily consolidated with spatial datasets.

Buchele et al. (2006) and Chen et al. (2009) discussed a modern approach for integrated flood risk assessment. In light of the setting of a more relative examination of different flood risk assessment models, for mapping, in the midst of extreme situations. The ampleness of synchronous and in-house proprietary methods using was analyzed by Chen and Zhan (2008). The study used an operator-based technique to model movement streams at the level of individual vehicles and examines the total practices of modeling and visualization of moving objects, during an emergency. De Silva (2000) presented a model Spatial Decision Support System (SDSS) which was normal for credibility making blueprints for emergency mapping, where response operations using spatial information dealing with and representation points of confinement in a GIS. It interfaces together with the geospatial part of the spatial analysis section is given by the GIS. The SDSS, so that gives a detailed spatial information of flood zone extension and involved layers.

Moreri et al. (2008) proposed an approach to manage making an internet-based Geographic Information Systems (WebGIS) application, which would reinforce people living in flood zones, which may at one point be unprotected in light of their closeness to the stream and the adequacy of the flooding. Zerger and Wealands (2004) showed that spatially quick hydrodynamic flood models could expect an essential part in average danger peril reducing. A key element of these models that make them suitable for risk exhibiting is the capacity to give time-blueprint immersion data about the onset, length, and embarking to an emergency situation. Such data can be the start for region utilize orchestrating, for mapping, for clearing directing, and for finding sensible crisis organization to give a couple of representations hazard responses. To address these confinements, a structure has been made that interfaces, with emergency response team with a GIS-based decision support system.

#### Dust storms

Dust Storms are otherwise called Sand Storms; it represents one of the common hazards with a broad range of environmental impacts. During an event of a stand storm, it affects human health in various ways. Sandstorms are a critical reason for car crashes and cause air transportation delays. Goudie (2008a, b) discussed the products during the process of stand storm eruption. It presents fine particles, salts and chemicals (counting herbicides) into the environment, with a suite of health effects, including respiratory complaints as well as different serious illnesses. Dust storms can transport allergens including microscopic organisms and growths, in this manner affect human health. Spatial Analysis can be exceptionally successful in displaying and representation the degree and the effect of sandstorms. Specifically, we can utilize GIS to give the accompanying capacities in managing dust storms disaster management.

The recent developments in global warming and climate change have prompted increased activity of sand storms in various parts of the word. Numerous researchers including Goudie (2008a, b; Xu et al. 2006) have dealt with the examination of sandstorms events and its impact on the land surface, utilizing GIS and Remote Sensing. Goudie (2009), discussed the first methodology relies upon the investigation of weather station information and representation of the spread of particulate matter in particular space in association with Dry Mid Temperature and Sub-Dry Temperature, particularly in the desert or semi-desert or zones. Measurable investigates exhibit that the event of sand–dust storms relate to a high degree of wind speed, which thus is firmly identified with land surface components; then again, a significant relationship between rain event and other atmospheric elements, for example, precipitation and temperature were not watched. This is notwithstanding the part of vegetation cover, which has been unequivocally connected to dust storms.

#### Health hazards

According to Cioccio and Michael (2007) Emergency management of health impacts, specifically focus on the vulnerable population; and access to medical services; GIS technology is capable in extreme heat attacks, by providing the degree and application for spatially analyzing the distribution of services and its relation to the population at risk. Despite all that, the literature that covers the use of GIS for health impact is somewhat limited. Many requests for the use of GIS in health focuses on the methodology, and the practical applications to the domains of vulnerable population, health care facilities distribution, and emergency shelters distribution. These three themes could be linked to the census and traffic information to provide more detailed spatial models, when dealing with this hazard.

Sharma et al. (2008) pointed out that one of the key utilizations of GIS in pandemic modeling and simulation is to encourage access to health services by inhabitants who live in and around the security area of a mass gathering or a social event. This will be achieved by outlining an application GIS to help health authorities in the planning and implementation of emergency medical response, with an emphasis on improving support of vulnerable population, including:

- Ensuring continuous routine for health services amid times of restricted access to a security area;
- Ensuring evacuation procedures for medical emergencies that are non-event related;
- Providing timely evacuation and health care in the event of mass causality incident.

This can be accomplished by outlining a mapping tool to locate vulnerable community members inside the affected zone, if there should arise an occurrence of a pending natural or technological disaster, for example, a heat wave or power outage. Becerra-Fernandez et al. (2008), explained that GIS could be used to for specifying access and evacuation routes, for approaching or in progress emergency or disaster management events. Goals may incorporate shelters, schools or other predefined destinations outside of the security zone Chandana et al. (2007). The key support of GIS in a pandemic episode can be through the utilization of GIS intending to general public health issues, particularly, to characterize its uses and restrictions in managing the inquiries of describing the vulnerable population. GIS supports advanced intervention operations, for example, Roland Daley et al. (2015) have highlighted some of these issues as following:

- Choosing sites for community flu centers and vaccination stations.
- Monitoring and assessing effect of vaccination centers and stations.
- Canceling public events, and gatherings.
- Closing schools, meetings and gatherings.
- Restricting utilization of public transportation frameworks.
- Identifying potential groups quarantine and isolation facilities.
- Enforcing people to follow group or individual isolates.

### Spatial analysis applications in technological hazards

#### Infrastructure disruption and malfunction

Cova and Church (1997) and Cimellaro (2016) discussed an approach for purposely recognizing neighborhoods that may go up against transportation challenges during an emergency evacuation. A description of this nature offers an interesting approach to manage assessing group of defenselessness in regions subject to advanced dynamic risks of uncertain spatial impact (e.g., hazardous spills on roadways). A heuristic estimation is delineated which can be usable for conveying useful, the excellent answer for this model in a GIS setting, as it was associated with a study region.

Camps (1993) presented a new computerized risk management framework for use by less experienced risk management personnel who to reduce the likelihood and seriousness of accidents. The framework, which was developed, is suitable for use in oil, gas, or chemical processing sites. It joins scientific models and calculation tools for accident simulation and building a database that incorporates accidents scenarios and response plans. It can likewise be utilized as a part of an emergency situation to decide favored approaches to find external assistance.

### Spatial analysis applications in manmade hazards

#### Mass gathering and civil unrest

Numerous sorts of mass gathering and the concentration of population change participating in such events may vary, depends on the nature of the event, its location and the time and season of the event. For instance, civil demonstrations, outdoor rock concerts, and a football match are typical examples where there is clear variation in the density of population attending these events. According to McDonald (2008), these occasions regularly, don’t draw in one sort of participants. Therefore, risks might be connected with weather related sickness, harmful impacts of medications, or injury because of members attempting to draw near to the stage. Bradler et al. (2008), concluded that political events, for example, political parties conventions might have several risks, associated with. This incorporate trauma or toxic impacts of depression related to a political protest or terrorism-related incidents. Becerra-Fernandez et al. (2008) have indicated that GIS spatial analysis is valid in this applications, as it provides:

1. Specifying the dissemination of individuals around the event proximity.
2. Analyzing the scope and approach for mapping evacuation if there should be an occurrence of an emergency.
3. Determining the positions and movement of law enforcement in the field.
4. Analyzing the pattern of development of masses.
5. Supporting effective decision-making on evacuation and response to an emergency situation.

#### Terrorism

Kwan and Lee (2005) have shown that the terrorist attacks on the World Trade Center (WTC) in New York City and the Pentagon on September 11, 2001, has not quite recently impacted multi-level structures in an urban center. They have also influenced by their surroundings at the street level in ways that reduced the time limits for the speed of emergency response. The capacity of using progressing 3D GIS for the headway and execution of GIS-based intelligent emergency response systems. The fact was at urging a quick emergency response to terrorist attacks on multi-level structures (e.g., multi-story office structures). A system design and a framework data show that facilitates the ground transportation capabilities with the inside courses inside multi-level structures into a protected 3D GIS was examined. Issues of using adaptable representation stages were also discussed especially the prerequisite for the remote and versatile response plan. Critical decision support functionalities were moreover considered with particular reference to the utilization of framework based most restricted way computations. A test use of expected 3D structure data shows a GIS database for a nearby study area was demonstrated by Kwan and Lee (2005). The study indicates that reaction delay inside multi-level structures can be any longer than deferrals caused on the ground transportation framework, have the potential for impressively decreasing these postponements.

Johnson (2003) demonstrated that in times of crisis, the disaster managers have the necessary commitment in regards to quickly and adequately managing any situation that may happen. An adjusted GIS application was delivered engaging a brief based examination of a catastrophe occasion facilitated with the centralization of masses distinguished correctly to the room level. The GIS Emergency Management System (GEMS) application is an astute structure to be utilized as a part of the Emergency Operation Center (EOC) to help the heading of the response. On the off chance that a calamity needs to happen, the intervention and recovery attempts could be at initially focused on the most fundamental areas of the greatest convergence of people.

## Challenges and trends

Goodchild (2006) indicated that various events, including the Indian Ocean Tsunami of 2005, the Hurricanes of the 2005 season, and the 7/7 and 9/11 terrorist’s attacks, have made each one of us seriously aware of the shortcoming of the modern society. Knowing the chronicled record of the events and where such situations have happened, notwithstanding the geographic limits of their effects are apparently essential, primarily when consolidated with data on human population and distribution, along with other spatially circulated wonders that might apply to reaction and recuperation (Abdalla et al. 2014). Regardless, GIS and spatial technologies that gather, analyze, and take into account visualization of such data, using advanced geomatics technologies in the form of GIS, remote sensing, GPS, and Photogrammetry. These technologies are unmistakably indispensable in all parts of the disaster management cycle, from protection, response, and recovery through acknowledgment to the reply and conceivable recovery. GIS give the preface to evaluating and mapping hazards, from evacuation planning to delivery to shelters, to routes planning to rehabilitation and restoration. Abdalla (2015) describes it as it likewise allows for choosing ranges where human population is well on the way to have been influenced by a disaster, and for allocating assets amid the recuperation procedure, among various other irreplaceable and crucial assignments that the GIS brings.

One of the distinct challenges in the utilization of GIS for urban disaster management is the location dependency of the event, or what is known as the geographic interdependence of the event. The proximity of the event can prompt many complexities in deciding the location, space and temporal parameters of the event. Various episodes in similar areas can bring about falling or raising impacts among the diversely affected entities.

As to the utility of Spatial Analysis in coordination approaches, the status of the spatial analysis confront noteworthy insufficiencies that can be condensed by the absence of some standard methods in a few districts and additionally the unstructured conventions that are being used when managing complex situations of disasters in various parts of the world. Some portion of this can be additionally ascribed to the absence of preparing in utilizing GIS frameworks, which can at times cause specialists on call for act wastefully when managing calamities. Spatial Analysis can bolster viable preparing for disaster management professionals to manage complex circumstances. More critically, the advocated requirement for geospatial information in a few parts of the world requires compelling access to worldwide SDI utilizing advanced access protocols and to strengthen the advancement of techniques and strategies for effective decision-making. This can be ascribed to the absence of interoperability in data exchange and processes standardization.

The decision-making process confronts a few difficulties on the lack of common operating picture for efficient policy implementation process. Decision-makers require to access spatial models actually in the form of real-time data feeds from planning teams, from field observers and remotely gathered information. This supports the viable access to operations areas monitoring devices, and as well it enhances the output of emergency management decision making.

## Summary

The spatial analysis gives powerful means in managing risk and in dealing with hazard mapping and assessment. It is likewise compelling in providing visual models that assist decision-makers with utilizing these advances adequately. However, spatial analysis can’t be satisfied without getting the exact information, defining proficient strategies and securing viable execution of HR required in Emergency Management.

The extent of this exploration is challenging, with an endeavor to cover a few risks that may cause disasters in urban regions. It is essential to deal adequately with emergency management in any of its four stages, i.e., preparedness, mitigation, response, and recovery. The discussion of the detailed application of spatial analysis overviewing the state-of-the-art application the technology has raised a few issues identified with the development of the utilization of spatial advances in disaster management, specific questions are:

1. The co-locality of an impact as a result of a series of events may require more progressed spatial analysis answers for giving details about the extent of damage, the cost of harm, the distribution of vulnerable population, the indicators of vulnerability and the mean for a response.
2. Issues with data and information frameworks of interoperability are essential in giving an available prearrangement through spatial analysis.
3. Health related emergencies are more complex to analyze spatially because of issues identified with private access to patient’s data, also the difficulty of covering various scales of events in the limited temporal timeframe.
4. Although the present GIS systems contribute to advanced spatial analysis capabilities; yet new methodologies for investigation, representation, and integration are required to offer additional means for support to urban disaster and emergency management community.

## Abbreviations

GIS: geospatial information systems
GEMS: GIS emergency management system
EOC: emergency operations centers
ESRI: Environmental Systems Research Institute
SDSS: spatial decision support system
SDI: spatial data infrastructure
GPS: global positioning system
3D: three dimensional
HR: human resources
